# Oral Immunotherapy for Food-Allergic Children: A Pro-Con Debate

**DOI:** 10.3389/fimmu.2021.636612

**Published:** 2021-09-28

**Authors:** Francesca Mori, Mattia Giovannini, Simona Barni, Rodrigo Jiménez-Saiz, Daniel Munblit, Benedetta Biagioni, Giulia Liccioli, Lucrezia Sarti, Lucia Liotti, Silvia Ricci, Elio Novembre, Umit Sahiner, Ermanno Baldo, Davide Caimmi

**Affiliations:** ^1^ Allergy Unit, Department of Pediatrics, Meyer Children’s University Hospital, Florence, Italy; ^2^ Department of Immunology, Instituto de Investigación Sanitaria Hospital Universitario de La Princesa (IIS-IP), Madrid, Spain; ^3^ Department of Immunology & Oncology, Centro Nacional de Biotecnología (CNB)-CSIC, Madrid, Spain; ^4^ Faculty of Experimental Sciences, Universidad Francisco de Vitoria (UFV), Madrid, Spain; ^5^ Department of Medicine, McMaster University, Hamilton, ON, Canada; ^6^ Department of Paediatrics and Paediatric Infectious Diseases, Institute of Child’s Health, Sechenov First Moscow State Medical University (Sechenov University), Moscow, Russia; ^7^ Inflammation, Repair and Development Section, National Heart and Lung Institute, Faculty of Medicine, Imperial College London, London, United Kingdom; ^8^ Research and Clinical Center for Neuropsychiatry, Moscow, Russia; ^9^ Allergy Outpatient Clinic, Division of Internal Medicine, IRCCS Azienda Ospedaliera Universitaria, Bologna, Italy; ^10^ Department of Pediatrics, Salesi Children’s Hospital, Azienda Ospedaliera Universitaria (AOU) Ospedali Riuniti Ancona, Ancona, Italy; ^11^ Division of Immunology, Section of Pediatrics, Department of Health Sciences, University of Florence and Meyer Children’s Hospital, Florence, Italy; ^12^ Department of Pediatric Allergy, Hacettepe University, Ankara, Turkey; ^13^ “Giovan Battista Mattei” Research Institute, Stenico, Italy; ^14^ Allergy Unit, CHU de Montpellier, Univ Montpellier, Montpellier, France; ^15^ IDESP, UA11, INSERM-Univ Montpellier, Montpellier, France

**Keywords:** food allergy, oral immunotherapy, IgE, reaction, anaphylaxis, pediatrics

## Abstract

The prevalence of food allergy has increased in recent years, especially in children. Allergen avoidance, and drugs in case of an allergic reaction, remains the standard of care in food allergy. Nevertheless, increasing attention has been given to the possibility to treat food allergy, through immunotherapy, particularly oral immunotherapy (OIT). Several OIT protocols and clinical trials have been published. Most of them focus on children allergic to milk, egg, or peanut, although recent studies developed protocols for other foods, such as wheat and different nuts. OIT efficacy in randomized controlled trials is usually evaluated as the possibility for patients to achieve desensitization through the consumption of an increasing amount of a food allergen, while the issue of a possible long-term sustained unresponsiveness has not been completely addressed. Here, we evaluated current pediatric OIT knowledge, focusing on the results of clinical trials and current guidelines. Specifically, we wanted to highlight what is known in terms of OIT efficacy and effectiveness, safety, and impact on quality of life. For each aspect, we reported the pros and the cons, inferable from published literature. In conclusion, even though many protocols, reviews and meta-analysis have been published on this topic, pediatric OIT remains a controversial therapy and no definitive generalized conclusion may be drawn so far. It should be an option provided by specialized teams, when both patients and their families are prone to adhere to the proposed protocol. Efficacy, long-term effectiveness, possible role of adjuvant therapies, risk of severe reactions including anaphylaxis or eosinophilic esophagitis, and impact on the quality of life of both children and caregivers are all aspects that should be discussed before starting OIT. Future studies are needed to provide firm clinical and scientific evidence, which should also consider patient reported outcomes.

## Introduction

The worldwide prevalence of allergic disease has increased over the last decades (1, 2). Among allergic diseases, food allergy (FA) represents a major public health concern as the leading cause of anaphylaxis in the pediatric population (3–6) and and being associated to a higher risk of severe forms in asthmatic children (7). Such assumption should be carefully taken into consideration because the prevalence of FA in children in Europe is ~3.1% (8) and more than a third of food allergic children have asthma (9). In addition, FA causes a considerable psychological impact both to the allergic patients and their families (10). For example, a pan-European study showed that most peanut-allergic individuals had lifestyle restrictions regarding food, faced problems with socializing, holiday activities and the use of public transport. Remarkably, two‐thirds of them felt socially isolated and over 40% had been bullied because of their disease (11). Furthermore, FA is an economic burden with an estimated household-level out-of-pocket equivalent to $3,339 and an individual-level direct medical cost of ~$2,081 worldwide (12).

Food allergen avoidance remains the backbone of the FA management (13–15). In the past years, extensive research has focused on intervention strategies to manage FA. The potential methods of allergen immunotherapy for FA include subcutaneous, sublingual, epicutaneous and oral immunotherapy. Moreover, combinations of immunotherapy with biologics, such as anti-IgE (e.g., omalizumab) or anti-IL4 receptor α (IL4Rα; e.g., dupilumab), or probiotics were proposed as well in the management of FA (16, 17). Of these, subcutaneous immunotherapy (SCIT) was popular in the 90s, but clinical trials were not successful due to the high frequency of systemic side effects (18, 19), which led to the use of hypoallergenic recombinant proteins (20). Although the use of SCIT in IgE-mediated FA has not been popular, it has gained more interest recently, using safer innovative research approaches both in patients and in murine models (21, 22). Nevertheless, SCIT is not currently used in routine clinical practice. Oral immunotherapy (OIT), followed by epicutaneous immunotherapy (EPIT), is the most studied intervention (13, 23). Indeed, peanut OIT and EPIT were recently approved by the Food and Drug Administration (FDA) for the treatment of peanut-allergic children (24). On the other hand, the implementation of OIT in FA management is generally debated (25).

OIT efficacy in randomized controlled trials (RCTs) is usually evaluated as the achievement of desensitization through the consumption of an increasing amount of a food allergen; this last is a state of increased allergen reactivity threshold as compared with the pre-OIT eliciting dose. However, it is unclear if desensitization or sustained unresponsiveness (SU), which is, in previously desensitized patients, the ability to safely consume any amount of the offending food, even after a prolonged period of allergen avoidance may be considered as the best outcome in the assessment of OIT efficacy/effectiveness (26). The same consideration may apply to the use of immunological parameters, in assessing efficacy and effectiveness, because their applicability as potential OIT outcome measures remains unclear.

Recent systematic reviews suggest that a number of OIT trials had methodological limitations. This fact not only may have led to an overestimation of OIT efficacy and an underestimation of reactions rate during OIT but could also be associated to an inaccurate representation of changes in health-related quality of life (QoL) in treated patients (27, 28). Furthermore, the lack of defined outcomes in FA intervention trials causes inconsistencies in terms of data interpretation (29). Additional criticisms towards OIT include discrepancies in decision-making knowledge on allergen dosing schedules, risk of secondary effects due to the procedure, questions on long-term clinical efficacy, cost-efficacy, and the burden of a potential daily treatment lasting over several years (25). On the other hand, some growing evidence in favor of OIT prompted active discussions on a possible wider introduction of OIT into routine clinical practice (30, 31). Indeed, many RCTs of milk, egg and peanut OIT were published. In this review, we critically appraise available scientific literature and, based on up-to-date evidence, provide arguments for and against OIT in FA children.

## Efficacy and Effectiveness

**Figure 1:**
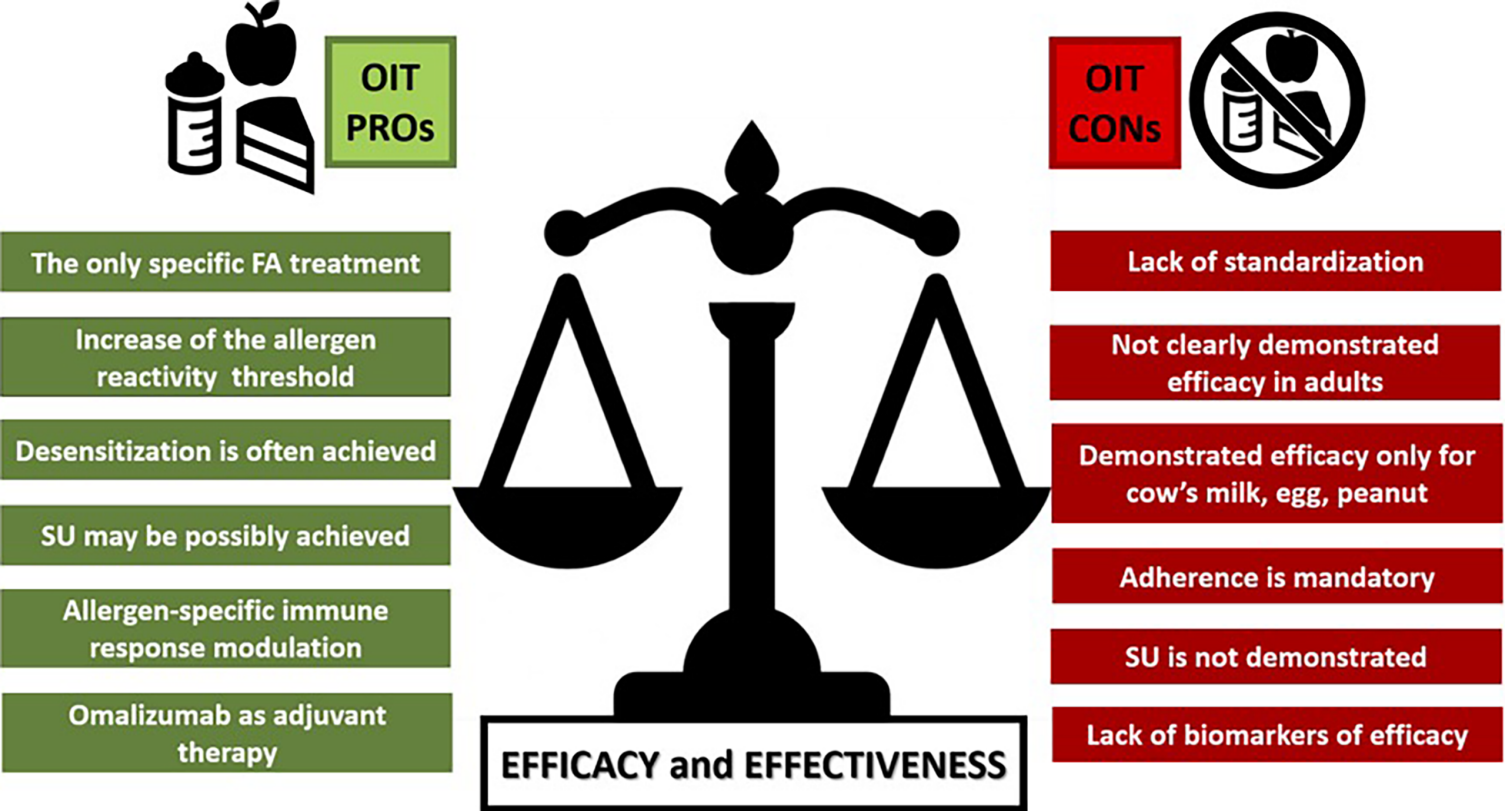
A comparison between the main PROs and CONs arguments on OIT Efficacy and Effectiveness. FA, food allergy; SU, sustained unresponsiveness.

**Table 1** and **Figure 1** summarize the pros and the cons with regards to efficacy and effectiveness of OIT in food-allergic children.

**Table 1:** Efficacy and effectiveness of OIT in children – pros and cons.

Efficacy and Effectiveness	
Pros	Cons
OIT increases the allergen reactivity threshold, providing protection in case of accidental allergen exposure Children can also suffer from persistent forms of FA, and OIT may be a specific treatment for this issue Most OIT-treated patients achieve desensitization The possibility of achieving SU increases if OIT is began in younger children OIT with some baked foods could accelerate tolerance acquisition towards uncooked foods and increase safety OIT modulates the allergen-specific immune response The use of omalizumab as adjuvant therapy for OIT facilitates reaching higher maintenance doses, over a shorter period	Studies on OIT are heterogeneous, hence it is difficult to assess evidence on its effectiveness In most cases, standardized products and OIT protocols are lacking There is no clear evidence of OIT efficacy in adults There is insufficient evidence of OIT efficacy for food other than cow’s milk, egg and peanut Children could spontaneously develop tolerance for certain foods (especially cow’s milk and egg), without the need of starting a challenging and time-consuming OIT protocol Patients’ adherence to treatment is critical for OIT success, and there are very few data on real-life experience confirming the results of published study protocols Accurate biomarkers of OIT efficacy are not available The combination of OIT and omalizumab does not seem to be associated with long-term tolerance achievement

A comparison between the main PROs and CONs arguments on OIT Efficacy and Effectiveness. FA, Food Allergy; SU, Sustained Unresponsiveness.

### Efficacy and Effectiveness – The Pros

OIT effectiveness is normally assessed using two possible outcomes: desensitization or SU (32, 33). As mentioned above, desensitization is the patient’s ability to increase the minimal amount of allergen required to elicit an allergic reaction; to be maintained, it requires daily allergen exposure (34). An allergen threshold increase provides a certain degree of protection on the accidental ingestion of the causative allergen (32). Moreover, some desensitized patients can regularly ingest a full serving dose without reactions. However, the ultimate OIT goal is the achievement of SU, which is, in previously desensitized patients, the ability to safely consume any amount of the offending food, even after a prolonged period of allergen avoidance (35). Desensitization does not preclude SU development. Indeed, desensitization is necessary to reach, afterwards, a state of SU.

It has long been established that 50% of cow’s milk allergic children and up to 80% of hen’s egg-allergic children develop tolerance by the age of 4-6 years (36–38). Moreover, recent studies showed that an increasing number of children tends to outgrow their cow’s milk and egg allergies after the preschool age (37, 39, 40). However, although FA often spontaneously resolves by preschool age, at least in patients allergic to cow’s milk and egg (41–43), the rate of patients outgrowing FA in adolescence is lower, with individuals suffering from persistent FA (37, 39, 40). Therefore, in these patients, immunotherapy represents a possible way to modify the natural course of their persistent FA.

A recent meta-analysis confirmed that OIT induces desensitization in most patients allergic to peanut, milk, and egg: 76.9% of OIT patients reached desensitization *vs* 8.1% of patients tolerating the foods following allergen avoidance or placebo (26). Although the capacity of OIT to induce SU has not been clearly demonstrated, this meta-analysis showed that SU developed in 31.8% of patients after OIT *vs* 11.1% after allergen avoidance or placebo (26). Of note, the likelihood of achieving SU appeared to be related to the duration of OIT. Indeed, 28% of egg-allergic patients reached SU after 22 months of egg OIT (35), compared to 50% after 4 years of treatment (44). Moreover, the rate of SU was higher in peanut-allergic patients when OIT was initiated at a younger age (9-36 months) (45). Although the ability of OIT to induce SU seems limited (26), in accordance to experts’ opinion, families may consider desensitization as an acceptable outcome, as it protects children from potentially severe allergic reactions due to accidental allergen exposure (46).

OIT efficacy/effectiveness is presumably dependent on its effects on the allergen-specific immune response (47, 48). The immunological basis underlying desensitization and SU during OIT are still poorly understood. This is in part due to the scarcity in studies on immunological mechanisms in OIT trials and to other possible limitations inherent to conducting research in FA pediatric patients (49). Notwithstanding, it is well established that OIT tends to reduce allergen-specific Ig (sIg)E (after an initial increase), and this is followed by a boost in sIgG_4_. The latter compete with sIgE for allergen binding, thus decreasing effector cell activation (*i.e*., basophils, mast cells), that release the mediators responsible for acute allergic reactions, including anaphylaxis (50). IgG_4_ has been the most studied sIg in OIT, but other subclasses may contribute to the overall blocking and inhibitory sIgG response in FA (50). Considering cellular immunity, the spotlight has mainly been on Treg cells, which typically increase during OIT and exert a beneficial, but transient, immunosuppressive function (48). Therefore, while there is evidence that OIT induces protective immunological mechanisms (47, 48, 50), our understanding of these circuits is still fairly limited, and therapeutic approaches to make them endure after OIT interruption remain to be clarified (49).

Recently, it has been shown that tolerance should not be considered as an “all-or-nothing phenomenon”. For example, some milk- and egg-allergic children tolerate baked forms of these foods (51, 52). This is due to the impact of heat-processing on the structure of immunodominant allergens of egg and milk (53, 54), which reduces the number of sIgE-binding sites (55, 56). Along this line, Esamaelizadeh et al. conducted a RCT in 84 children presenting with cow’s milk allergy but tolerant to baked milk. Patients were divided into a case (baked milk consumers) and a control group (baked milk avoiders) for one year: 88.1% of patients in the case group and 66.7% of those in the control group developed tolerance to unheated milk at the end of the study (57). Therefore, the use of cow’s milk as part of baked food in OIT may increase safety and may favor the resolution of allergies towards the native allergens (raw or unprocessed) (58–60). Nevertheless, further studies are needed to confirm this line of evidence and to ascertain involved mechanisms (57, 61, 62).

In addition to the use of baked or hypoallergenic foods, other approaches have been explored to improve OIT efficacy. One of them included the use of anti-IgE monoclonal antibodies as adjuvant treatment for OIT patients (63). Omalizumab was first used in combination with OIT in milk-allergic children (64). Subsequent studies showed that omalizumab facilitated a faster achievement of higher OIT maintenance doses as compared to regular OIT (32, 65–69). Importantly, while omalizumab used as an adjuvant to OIT improved its safety and tolerability, it did not lead to higher efficacy (67), although larger studies are required on this topic (70). In this context, biologics intended to block other pathways of the Th2 immune response, such as dupilumab have begun to be explored in FA patients (71). The idea of interfering with IL-4Rα, thus blocking IL-4/IL-13 signaling, would prevent IgE re-generation from any memory B cell reservoir that requires IgE class-switching and its commitment to a plasma cell lineage (49, 72, 73); in addition, *de novo* Th2 polarization would be hampered. Consequently, the concomitant interference with IL-4 and IL-13 signaling in FA, may not only impair the machinery re-generating IgE but also potentiate regulatory pathways leading to desensitization, SU or oral tolerance (72, 74, 75).

### Efficacy and Effectiveness – The Cons

Even though OIT has been evaluated for different allergens through several trials, in most cases this approach still lacks standardized protocols and current evidence has been generated only in a selected proportion of pediatric FA patients and only for certain foods (20). As abovementioned, OIT efficacy in clinical trials should clearly distinguish between desensitization and SU. Furthermore, patient-reported outcomes measures should be included in the clinical trials as well, because they represent powerful and irreplaceable tools to quantify the patient’s perception of the disease status and of its improvement (32).

OIT trials have shown effective desensitization in many patients and SU in some, but it is still unclear if the most relevant and important outcomes were measured. At present, there is no consensus on the core outcome domains, and validated instruments to assess these domains are lacking as well (29). The heterogeneity in OIT products, protocols, outcomes, age and clinical features of the enrolled patients does not allow to adequately assess the treatment effectiveness (27). Some preliminary estimations may be drawn for certain FA (e.g., cow’s milk, egg, and peanut). However, these only apply to children, because OIT in adult patients appears to not lead to successful desensitization (26). Importantly, children have a considerable likelihood of spontaneously acquiring tolerance, particularly to cow’s milk and egg, which questions the utility of OIT in them (76). Therefore, in some cases, it may be more appropriate to wait for the natural development of tolerance before proposing such a challenging and time-consuming intervention. On the other hand, those children that do not spontaneously outgrow their FA and become adults, may have lost a sensible window to modify their hypersensitive immunological status.

Another line of criticism towards OIT considers that the evidence in support of OIT efficacy in FA is weak and relying on limited data for cow’s milk, egg, and peanut. At present, OIT is not recommended for many foods, neither in adults nor in children (20). Most OIT clinical trials assessing effectiveness do not consider adherence problems although such issues remain critical in real-life settings. Indeed, OIT is a very demanding therapeutic option, and its efficacy strictly depends on patients’ adherence to treatment (20). In addition, consistent clinical and laboratory data on SU are scarce (77) and OIT is currently not widely used in the adult population worldwide. SU may be clinically confirmed by performing a food challenge after OIT has been discontinued for a certain period of time. In most studies, SU has not been assessed and there is not enough information on the possible efficacy of OIT in acquiring it after treatment discontinuation (26). Moreover, there is no consensus on when SU should be assessed, especially on how much time after OIT cessation. The majority of studies addressing SU evaluate it up to 8 weeks after treatment discontinuation (34), which may be insufficient to reach a firm conclusion. Furthermore, even once SU is confirmed, it may be lost over time. For example, it has been demonstrated that after a 2-weeks proven SU, patients treated with OIT for cow’s milk, egg and wheat may still experience allergic clinical manifestations after longer periods of avoidance (78). The lack of evidence for SU implies that OIT patients should pursue a life-long “maintenance phase” to prevent potentially dangerous adverse reactions (ARs) after the consumption of the involved food. To date, protocols do not include accurate information on possible quantity and frequency of food intake after the end of the maintenance phase. Therefore, in most cases there is a lack of standardization and of recommendations on this aspect of patients’ after-treatment management.

Several immunological changes have been reported during OIT, some of which appear to be consistent across different allergens and OIT protocols (50). As indicated earlier, OIT studies usually show an increase in sIgG_4_ levels, and its persistence upon therapy cessation has been associated with SU (79). Other immunological changes reported during OIT include expansion of Treg cells, and reduction in total and sIgE levels (47, 50). However, reliable biological markers to assess the evolution of OIT patients or desensitization/SU persistence are still unavailable. The suppression of the immunologic response during OIT seems to be transient (80, 81) and unable to control persistent populations of pathogenic Th2-cells, which have been detected in patients with peanut allergy after 12 to 24 months of OIT (80). Hence it is unclear if immunological markers can serve as a practical and reliable outcome of OIT (48). A detailed characterization of the immune cells affected by OIT is required to uncover immunological changes indicative of durable SU or effective desensitization, which can be tracked after OIT discontinuation (48, 49, 77). In this context, reaching a better understanding on the immunological basis of persistent FA may lead to the identification of novel biomarkers that may help in better defining OIT outcomes (49, 72–74).

The use of omalizumab as adjuvant therapy for OIT showed some benefits in facilitating a faster achievement of higher maintenance doses, as discussed above. However, omalizumab failed to improve SU acquisition. This may be potentially connected to the fact that the benefit obtained is lost after the discontinuation of the treatment with this monoclonal antibody (69).

At present, OIT may be considered for recommendation only in cow’s milk, egg or peanut allergic children and desensitization is assured only if the adherence to the treatment is very high. In most cases, appropriate products and defined protocols for OIT are still lacking. OIT to baked foods may be particularly challenging in centres lacking expertise. To date, effectiveness in inducing SU has not been demonstrated neither clinically nor in terms of persistent immunological modifications. Therefore, post-desensitization strategy remains unclear and further studies are needed. New approaches such as the utilization of omalizumab to improve OIT effectiveness failed to achieve long-term SU. Of note, lack of agreed core outcomes in FA slows down the process of high-quality evidence collection and OIT effectiveness assessment.

## Safety

**Figure 2:**
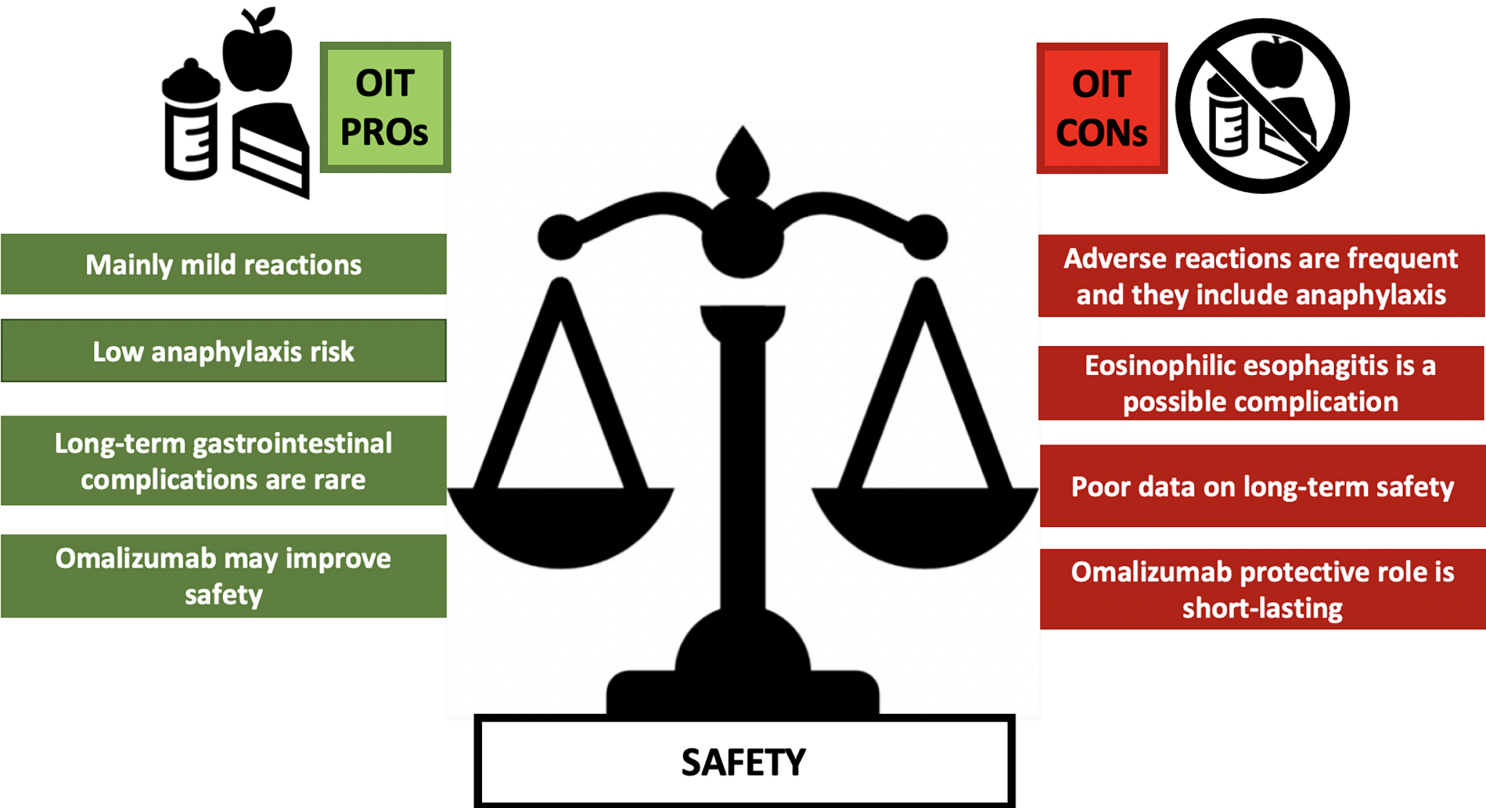
A comparison between the main PROs and CONs arguments on OIT Safety. ARs, adverse reactions; EoE, eosinophilic esophagitis; GI, gastrointestinal.

**Table 2** and **Figure 2** summarize the pros and the cons with regards to safety of OIT in food-allergic children.

**Table 2:** Safety of OIT in children – pros and cons.

Safety	
Pros	Cons
Most ARs reported during OIT are mild and easy to treat The overall risk of severe anaphylaxis is low Long-term gastrointestinal complications are rare and can resolve Omalizumab could be considered a useful OIT adjunct to reach a maintenance dose improving safety in severe FA patients	ARs, including anaphylaxis, occur mainly during the build-up phase. EoE is a possible complication of OIT and it may be underestimated Many cofactors may determine ARs, and require dose-adjustments Data on long-term safety are insufficient Decreasing omalizumab doses is related to an increased risk of ARs

A comparison between the main PROs and CONs arguments on OIT Safety. ARs, adverse reactions; EoE, eosinophilic esophagitis; GI, gastrointestinal.

### Safety – The Pros

FA patients and their relatives should be always aware of the potential risks associated with OIT. Various studies assessed OIT safety comparing intervention data with patients on elimination diet. Almost all OIT patients experienced mild or moderate ARs (46), as perioral rash, local urticaria, rhinitis, or minor gastrointestinal clinical manifestations (26), and most ARs resolved without treatment or simply after administration of oral antihistamines (82).

The risk of systemic ARs during OIT is relatively uncommon, and no OIT-related deaths were reported in the literature. Still, all FA patients should be trained to use, and carry, emergency drugs (epinephrine auto injector), and should be accurately monitored, especially in the OIT up-dosing phase (83). A recent baked-milk OIT study reported ARs in 21 out of 63 patients, with only one of them developing anaphylaxis (84). Other studies confirmed a low incidence of ARs requiring epinephrine injection during peanut (30, 85) and egg (86) OIT. In contrast, Kauppila et al. reported that 14% of patients had anaphylaxis during raw milk OIT, but a cofactor was often considered as an element involved for inducing the AR, or the ARs appeared after a period of allergen elimination diet (87).

Cofactors may play a role in triggering an AR, which mainly occurs during the OIT build-up phase. Importantly, patients already tolerating a specific allergen dose, may sometimes experience an AR during the OIT maintenance phase. In those cases, a cofactor altering immune homeostasis (e.g., viral infections, fever, exercise, non-steroidal anti-inflammatory drugs intake, or hormonal changes) may be involved (46). Indeed, it is important to recommend avoidance of physical activity at least one hour before and three hours after the intake of a food allergen. Likewise, children suffering from fever or infectious diseases should suspend, or at least halve, the OIT maintenance dose for a few days (82). Other conditions contributing to ARs during OIT are poorly controlled asthma, seasonal pollen allergy, and circumstances such as consumption of the OIT on an empty stomach (82).

Once long-term secondary effects are concerned, it is still debated whether OIT might play a role in the onset of eosinophilic esophagitis (EoE) (82, 88, 89). A meta-analysis reported a not negligible prevalence of EoE in OIT-treated patients (90). However, EoE is a condition that can resolve following specific therapy or OIT discontinuation (89). Along this line, the results of a recent trial on 15 adults undergoing peanut OIT showed that possible OIT-induced EoE and gastrointestinal eosinophilia are usually transient and not always associated with gastrointestinal signs and symptoms (91). Recurrence of ARs may also be a cause of OIT withdrawal (92, 93). A recent meta-analysis reported an overall rate of OIT discontinuation of about 14%; only 4.7% was due to clinical manifestations possibly related to EoE (92). In addition, Blumchen et al. showed that, using a peanut OIT protocol with a low maintenance dose, the proportion of dropouts due to ARs was 6.7%, without need of epinephrine use and with no EoE development (31). Nevertheless, it is still unclear if OIT can induce EoE as a direct side effect of the treatment.

The decision to initiate OIT should be tailored on the patient’s allergic profile, and on personal choice of patients and their families. Awareness of the possible risks should be raised, and details of the heterogeneity of reported ARs should be clearly outlined and put into context of the specific treatment proposed (88, 94).

### Safety – The Cons

Patient safety is a critical issue for OIT. ARs, including life-threatening events, appear to be more frequent during OIT, in comparison with food allergen avoidance (28, 88, 89, 94). Studies report that 10–35% of FA children withdrew OIT trials because of anaphylaxis, acute or repeated ARs and especially chronic abdominal pain (95, 96). As mentioned above, many identifiable but often unavoidable factors may cause ARs (including fever or infections, exercise, temperature changes, dosing on an empty stomach, menstruation, seasonal allergies, asthma, and non-compliance), and some identifiable cofactors are often unknown (28, 82, 97, 98). For example, near-fatal reactions have been observed in asthmatic teenagers showing poor compliance (99). Therefore, the presence of cofactors requires frequent allergen dose adjustments to obtain a safe dosing profile (100). In this regard, the European Academy of Allergy and Clinical Immunology (EAACI) OIT guidelines suggest continuous surveillance of OIT patients for ARs and clinical manifestations of new-onset EoE, especially during the up-dosing phase (20).

EoE is a possible secondary effect of OIT, but the relationship between EoE and OIT remains controversial, being unclear whether OIT causes EoE or reveals a pre-existing condition (89, 101). The overall prevalence of EoE following OIT was reported as 2.7% (82, 90). However, a prospective food OIT study reported that EoE or eosinophilic gastroenteritis developed in 7 (7.2%) patients out of 97 children included in a milk OIT group and in 2 (6.4%) out of 31 patients in a egg OIT group (102). During OIT, sIgG and sIgA may enhance eosinophil activation and contribute to EoE onset (103). To evaluate the real EoE prevalence during and following OIT, it is necessary to consider that an esophagogastroduodenoscopy is not performed in all patients with dose-limiting gastrointestinal clinical manifestations. Therefore, the real EoE rate may be higher than reported (104). Furthermore, longitudinal data of EoE in food OIT are insufficient to study long-term safety (93). A recent comprehensive review analyzed data from 110 studies (92) and found that EoE-confirming biopsies were performed in 18 studies only, and EoE was diagnosed in 5.3% of OIT patients.

Many studies show that epinephrine use is variable and related to the OIT protocol (105). Wasserman et al. retrospectively reviewed charts from 352 patients evaluated in 5 allergy centres, and they found that epinephrine administration was necessary in 36 patients (10.2%) (106). A Cochrane systematic review on milk OIT reported ARs in 97 out of 106 patients (91%), while epinephrine was required in 9% of patients receiving milk OIT (107). A more recent prospective study on milk OIT registered 1,548 ARs, most of which occurring during the escalation phase (89.6%). Anaphylaxis and chronic late-onset gastrointestinal clinical manifestations accounted for 15.8% of ARs and represented the primary reason for protocol withdrawal. Interestingly, a higher rate of sIgE for α-lactalbumin and casein at baseline was associated with an increased risk of anaphylaxis during milk OIT, while patients with higher sIgE for β-lactoglobulin had a lower risk (108). Moreover, egg OIT was associated with serious ARs in all 10 RCTs included in a Cochrane systematic review. Epinephrine was required in 21 out of 249 (8.4%) of children in the egg OIT group but never in the control group (105). A recent systematic review of >1,000 peanut-allergic patients evidenced that peanut OIT increases the risk of ARs, anaphylaxis development and epinephrine use, either during the build-up or the maintenance phase. The authors estimated the risk of anaphylaxis in patients undergoing OIT about 22% in comparison with a baseline risk of 7% (28).

Some centres are using omalizumab during OIT because it may reduce the risk of ARs, especially in children with severe FA. However, the duration of the therapy with omalizumab is still debated, as well as the long-term outcomes after omalizumab discontinuation (66). Some experts consider the use of omalizumab as a helpful measure for rapid up-dosing but, when decreasing omalizumab doses, ARs become more frequent. Therefore, omalizumab appears to confer short-term protection from OIT-related ARs (109). Furthermore, the use of omalizumab may be associated with side reactions including skin inflammation and anaphylaxis in 0.1-0.2% of patients, likely due to its engagement with Fcγ receptors (110).

Further investigations on OIT safety should be carried on prior to routine OIT use in clinical practice (105, 111). A careful and complete explanation of the ARs risks *vs* OIT clinical benefits should be given before starting the therapy to both children and their families (83).

## Impact on QoL

**Figure 3:**
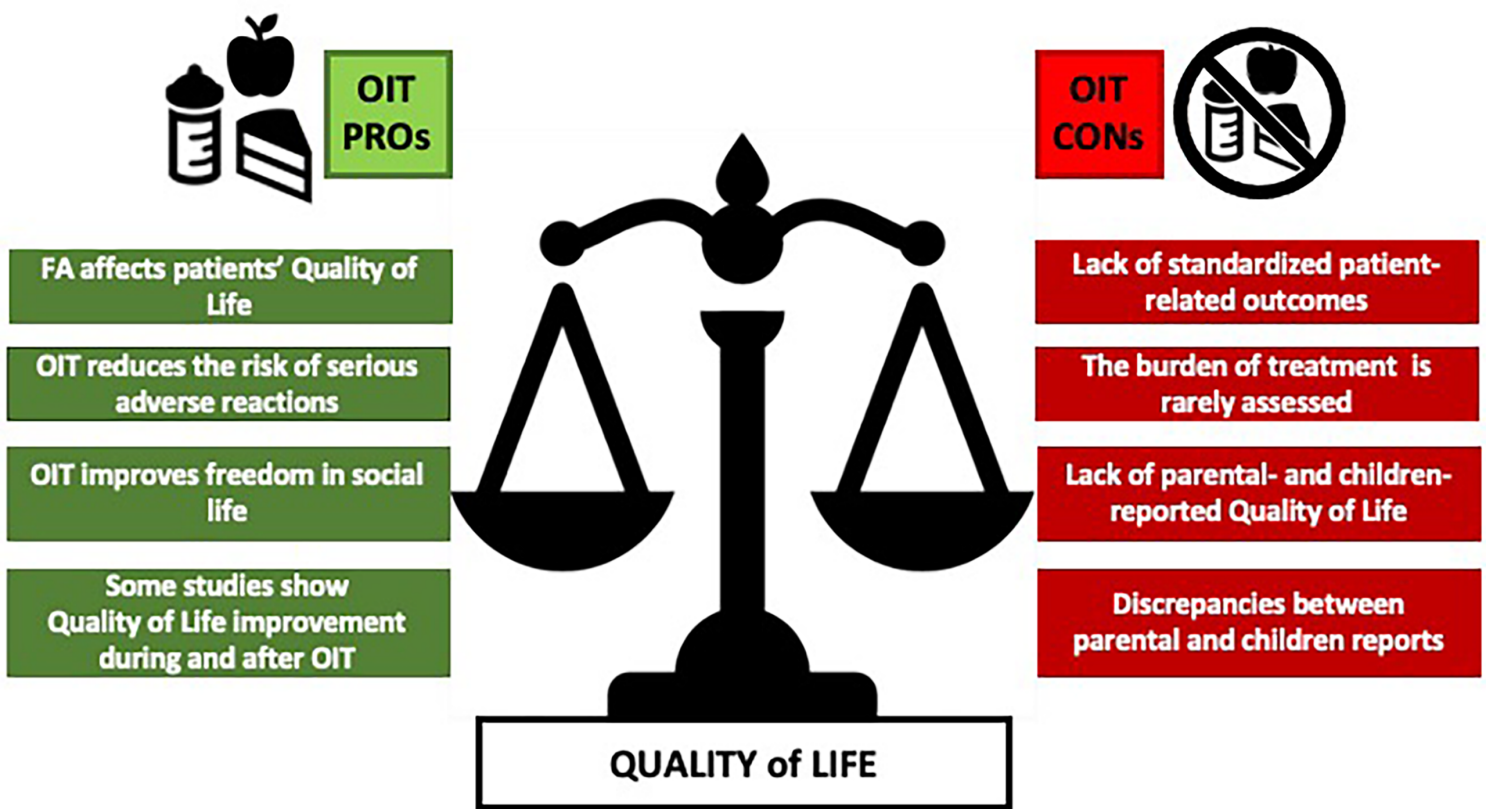
A comparison between the main PROs and CONs arguments on OIT quality of life. FA, food allergy.

**Table 3** and **Figure 3** summarize the pros and the cons with regards to the impact on QoL of OIT in food-allergic children.

**Table 3:** Impact of food OIT in children’s QoL – pros and cons.

QoL	
Pros	Cons
FA affects patients’ QoL due to food-related anxiety, fear of accidental exposures and the ever-present burden of social and dietary limitations Desensitization may reduce the risk of serious reaction after accidental allergen exposure Desensitization would lead to greater freedom in the social life of FA patients and consequently less fear in their daily life Some studies showed an improvement in QoL during and after OIT	Validated and uniform patient-related outcome measures have been rarely used in OIT trials OIT burden of treatment has been rarely assessed, and it could have a negative impact on patients’ QoL Considerable discrepancies emerged between children’s and parents’ reports on QoL OIT may be associated with a worsening in QoL, during the build-up phase, especially because of the appearance of possible ARs.

A comparison between the main PROs and CONs arguments on OIT quality of life. ARs, adverse reactions; FA, food allergy; QoL, quality of life.

### Impact on QoL – The Pros

Both FA and the consequent elimination diet have a negative impact on QoL due to food-related anxiety, fear of accidental exposures and the ever-present burden of social and dietary limitations (11, 95, 112–114). Allergen elimination diet remains the gold standard for FA management. This approach, however, requires constant responsibility from both patient and caregiver. FA children are vulnerable to unintentional allergen ingestion and possible anaphylaxis, which has a negative influence on the QoL (35).

OIT aims to increase the allergen reactivity threshold to reduce the risk of serious allergic reactions after inadvertent allergen exposure. This approach should provide a safer social life for FA children, with less fear of being accidentally exposed to the allergen, and eventually result in QoL improvement. A complete resolution of the allergy following OIT treatment remains the ultimate OIT goal, which would clearly improve patients’ QoL. An increasing number of studies evaluated the impact of OIT on children’s QoL, mainly for egg (115, 116), peanut (11, 31, 96, 117–119), and cow’s milk allergy (120), but also on patients suffering from multiple FA (121–124). The results of these studies are encouraging, even though most of them report major limitations mainly related to the small sample included in the study and the limited number of RCTs.

Most of the studies confirmed that desensitization correlates with an improvement in children’s QoL, as perceived by their caregivers (96, 115, 121, 122, 124, 125). Three factors were found to be associated with a more substantial improvement in QoL: having an allergy to a single food, presenting with a history of anaphylactic reactions prior to OIT initiation, and having a very low QoL before the beginning of OIT (124). Blumchen et al. found that achieving desensitization during OIT improved the QoL as perceived by caregivers and by patients in a double-blind RCT (31). After peanut OIT, there was a significant improvement in QoL in the domain of “risk of accidental exposure” and “emotional impact” in children, when compared with the placebo group. Other studies that evaluated the QoL during and/or at the end of OIT showed a more substantial improvement in children (116, 117) or adolescents (117) when compared to the caregiver-reported QoL. In contrast, Reier-Nielsen et al. found statistically significant improvements in QoL reported by caregivers, while no significant change was recorded in children (119).

Several studies evaluated QoL improvement by OIT, as perceived by caregivers, in patients achieving SU (11, 120, 126). Specifically, a double-blind placebo-controlled RCT (127) evaluated the impact of Probiotic and Peanut Oral Immunotherapy (PPOIT) on health-related QoL. The authors concluded that PPOIT had a sustained beneficial effect on the psychosocial impact of FA at 3 months and 12 months after completion of the treatment. The improved QoL was specifically associated with the acquisition of SU. Indeed, for this study, a *post-hoc* analysis revealed that no improvement in QoL was seen for either PPOIT-treated or placebo-treated patients who failed to achieve SU (128). A study assessed the impact of QoL evaluated by children and adolescents after achieving peanut SU (126); in this study, there was a general trend towards an improvement in QoL at the end of peanut OIT, but it did not reach statistical significance, probably because of the small sample size.

Studies assessing QoL as one of the outcomes in patients undergoing OIT are heterogeneous, use different methods, and are often limited to a small sample size. According to some experiences, compared to food allergen avoidance, OIT may be associated with an improvement in QoL for both patients and caregivers, especially at the end of the treatment and in the absence of ARs (122, 129). However, these data should be viewed with caution as additional evidence is needed.

### Impact on QoL – The Cons

FA patients present with a decreased QoL, with repercussions on their general health and lifestyle (20, 130). Although OIT is a promising therapeutic approach, it is demanding, especially in terms of protocol duration, adherence-related issues, and safety concerns (20). It is therefore evident that improvements in patients’ QoL throughout and after OIT must be carefully assessed (26, 131). The heterogeneity of factors possibly influencing QoL makes it difficult to achieve a uniform approach. Some important ones such as the perception of treatment burden, are rarely considered in OIT trials (31). Health-related QoL has only recently started to appear as a potential outcome in OIT clinical trials, hence data on the matter are limited (131) and a meta-analysis by Chu et al. found no evidence that OIT improves the QoL (28).

Improved QoL after OIT has been reported only in a few trials for egg (115, 116) and cow’s milk allergy (120), while a larger number of studies considering QoL is available regarding peanut (31, 96, 118, 119, 126, 127) and multiple food desensitization (121, 122, 124, 125). Most of these studies are characterized by substantial limitations in the reported results, being based only on parent-reported QoL (96, 121, 123, 128) or lacking a control group (117). As aforementioned, discrepancies between children self-assessment and parental reports have emerged, showing significantly better QoL scores reported by parents when compared to their children (119). These data suggest that parents may overestimate the impact of OIT on child’s QoL, calling into question the appropriateness of parental proxy reports use as a valid outcome of OIT effectiveness. In addition, OIT may also result in QoL worsening, for example in the build-up phase, as demonstrated by Ebstein-Rigbi et al., probably because of the occurrence of ARs in this treatment phase (122). The heterogeneity of the studies and of the methodology of QoL assessment, along with the very limited number of data do not allow for definitive conclusions but the most up to date meta-analysis does not add optimism (28).

## Discussion

FA is one of the most burdensome allergic disease in children (132). It affects both patients and caregivers, as there is always a risk of accidental exposure, and they should be ready to administer a treatment for the clinical manifestations, in case of a reaction, with the consequent impact on their daily life. Although food allergen avoidance and treatment of the signs and symptoms are still the mainstream of FA management, OIT has been proposed as an alternative approach aiming at the “disease treatment”, focusing on its natural history. Well-designed OIT clinical trials were initiated recently, making OIT a considerably novel approach in FA management; nevertheless, the first attempts of treating children by giving small and increasing amounts of the allergen, to stimulate immune tolerance, go back to the beginning of the 20^th^ century (133). Most OIT trials were conducted over the last decade focusing on single allergens, predominantly on cow’s milk, egg, and peanut, but there is an increasing number of publications on other allergens, such as wheat (134–136) and nuts (137). Moreover, peanut OIT and EPIT were recently approved by the FDA for the treatment of peanut-allergic children (24). The novel approach of treating children suffering from multiple FAs with OIT is showing some promising results (138, 139), which is of a particular interest to practicing physicians as it is closer to routine clinical practice settings.

There is an intense debate over the utility of OIT in children: there is a substantial heterogeneity of study protocols with regards to administration schedules (*e.g.*, starting doses, doses increase, delay between doses, and target maintenance dose); and they diverge on efficacy and safety profiles, as well as on QoL assessment in the treated population. Here, we reviewed existing evidence on this subject, highlighting discrepancies and summarizing the main findings. OIT is capable of increasing the allergen reactivity threshold dose. Although desensitization seems to be beneficial for the FA patient, the extent of this improvement is unknown as no agreement on core outcomes is in place (29). FA patients are facing the constant fear of developing a reaction after inadvertent consumption of the causative allergen, which remains one of the major issues for them and their caregivers. Increasing the allergen eliciting dose reduces, to some extent, the risk of allergic reactions, thus decreasing stress in FA patients and their relatives. While desensitization is an achievable OIT outcome, SU remains a distant prospect. In addition, studies thoroughly assessing SU are scarce and mainly focused on short-term effects.

The use of adjuvant therapies, as an OIT adjunct, may empower the effects of OIT. Adjuvant therapies currently under investigation include toll-like-receptor 4 and 9 agonists; nanoparticles encapsulating the allergen; Chinese medicine; antihistamines; leukotriene receptor antagonists; probiotics; and biologics, especially omalizumab and dupilumab (71, 140). The combination of OIT with omalizumab is believed to decrease the risk of ARs and/or allow faster desensitization with a better safety profile (66, 141).

A recent observational study assessing the effects of omalizumab in 15 asthmatic children showed 8.6-fold increase in food allergen threshold (142). The concomitant use of omalizumab during OIT seems to promote allergen desensitization through an initial omalizumab-dependent step that acutely depletes allergen-reactive T cells. This appears to be followed by an allergen-specific Treg cell activity increase due to the reversal of their Th2 cell-like program. Improved Treg cell function could therefore be the mechanism allowing an easier and faster OIT protocol in FA patients (143). Preliminary results are encouraging, but optimal duration of omalizumab use before/during/after OIT, and its role in the treatment of FA remain unknown. More clinical trials are therefore needed to determine the patients’ phenotype that is suitable for biologics therapy and optimal treatment regimens. A currently ongoing blinded study is comparing OIT with and without omalizumab (138). The results will possibly allow to single out the effect of omalizumab on OIT effectiveness and safety, hence providing novel prospective data to inform on the optimal and most cost-effective dosage for this indication (138). Lastly, the therapeutic value of dupilumab has begun to be assessed in FA patients (71). The blockade of IgE class-switching from IgG^+^ memory B cells and preventing the perpetuation of the Th2 program during OIT may yield safer therapies and favor the transition from desensitization to SU or even oral tolerance (72, 74, 75).

OIT safety has not been clearly determined. Most ARs occurring during the build-up phase are normally mild to moderate, but concerns remain regarding less frequent but potentially life-threatening ARs during OIT. Most studies report the occurrence of anaphylactic cases, especially during the OIT build-up phase. Furthermore, anaphylaxis is more common in patients undergoing OIT when compared with the control group, in clinical trials. It should be noted, however, that research settings may differ from the “real life scenario” as patients included in a study tend to meticulously follow investigator advice. Thus, studies evaluating the risk of anaphylaxis in “real-life settings” are needed. There are safety concerns due to the limited data and major methodological discrepancies between the trials and real-life populations in which patients having experienced anaphylaxis or suffering from severe asthma could be found. Indeed, although was shown that severe anaphylaxis is mainly associated with peanut allergy in children, most studies consider a history of severe anaphylaxis (or of repeated anaphylaxis episodes) (31, 65, 144–147), severe asthma (31, 65, 144–149) as an exclusion criteria. A limited number of trials did not exclude children with a history of severe and/or non-controlled asthma (96), or severe or repeated anaphylaxis (96, 148, 149). They did not find an increased risk of systemic/severe ARs in treated patients. Notwithstanding, the available data are not sufficient to establish an OIT safety profile in these particularly vulnerable groups of FA patients.

Because of the substantial heterogeneity of published studies, it is not possible to clearly define all the OIT pros and cons. ARs from OIT may be provoked by a wide range of factors including exercise, menses, colds, fever, alcohol, nonsteroidal anti-inflammatory drugs and other medications (46). Moreover, the frequency of ARs may affect OIT compliance resulting in patients to stop OIT over time. Another major problem is the absence of specific biomarkers predictive of OIT outcomes. Discussion with patients and their families, and meticulous explanation of the procedure including possible ARs, and the expected goals is pivotal. OIT outcomes should also be discussed and carefully analyzed by clinicians, patients, and their families together. An appropriate patients’ selection represents a cornerstone to increase the treatment’s probability of success and adherence. The latter choice appears really difficult, and it involves medical (*e.g.*, sensitization, history of reaction) but also human factors, especially related to the estimated compliance of the patient/family.

The main OIT aim could be a decrease in the risk of ARs to trace contamination or accidental exposure, or introduction of the food to the regular diet. Importantly, patients and families should be well informed of the duration of maintenance. It is crucial to understand that this type of treatment is associated with daily ingestion of a given food for years, and consequent limitations, particularly related to physical activity and other cofactors. Furthermore, they should be aware of the prospect of not reaching SU. The food and the appropriate age for OIT initiation must be carefully considered, taking into account that most children eventually acquire natural tolerance to cow’s milk and hen’s egg. In some cases, it may be more appropriate to wait for the natural tolerance acquisition; on the other hand, the risk is that children who do not spontaneously outgrow FA may have lost a suitable window of OIT intervention.

One of the main OIT limitations is the lack of solid data on long-term QoL improvement in OIT patients and lack of consensus on what (and how) OIT outcomes should be assessed in OIT trials (29). OIT safety and efficacy may be dependent on the age of the patients, and some studies have suggested that desensitization in younger children may be associated with better outcomes (30). However, it is exceedingly difficult to confirm whether desensitization or SU are the result of OIT or the natural resolution of FA in some cases in this age group.

A final aspect on which the present review did not focus, due to the lack of substantial literature on the topic, is the cost-effectiveness of OIT in FA patients. OIT has already turned into a treatment for FA in some countries and potential for OIT to be cost-effective and cost-saving should be assessed in detail prior to a wider implementation.

As for today, many aspects of FA management do not seem cost-effective, such as not being able to provide a short delay for OFC for eligible patients, or not having good biomarkers to firmly diagnose FA without the need to perform an OFC (150). To affirm that OIT is cost-effective, there are 3 levels that need to be taken into account: patients’ health state utility improvement, reduction of the risk of anaphylaxis, and likelihood of achieving SU. It should also be considered the degree to which patients will perceive the benefit of OIT (e.g., depending on the level of protection from accidental exposure; the possible anxiety reduction in patients and caregivers; and the possibility of making dietary changes after a successful therapy) (151).

Two recent papers highlight the fact that peanut OIT (with a commercial product) shows more favorable cost-effectiveness, compared to avoidance, with greater improvements in health utility, particularly if SU can be achieved (150, 151). Further studies are needed to determine the degree of health state utility improvement, and whether OIT will induce durable SU, allowing discontinuation of OIT (150, 151). Also, identifying patients who poorly respond to OIT would prevent continuing the treatment, but more studies are essential to better understand the predictive capacities of food immunotherapy biomarkers, and longer-term data will contribute to reach more solid health and economic analyses (151).

In conclusion, OIT remains a controversial treatment option, requiring team decision making, with patient, family, and physician involved, and all potential risks and benefits should be reviewed. This therapeutic approach has benefits, and it is associated with effective results in terms of increasing the eliciting dose of allergens in many patients, but it also carries significant risks, such as a higher rate of ARs than patients following strict allergen avoidance. Moreover, the impact of OIT on patient-reported outcomes, including QoL remains an open question and it should be assessed in future studies. Further research may help to improve the safety and efficacy of OIT as well as to identify patients who will benefit the most from OIT and will experience minimal ARs. Emerging data from clinical trials suggest that food OIT is a promising treatment modality, which provide patients and their families with an alternative to allergen avoidance and use of rescue medications. However, OIT should be an option provided by specialized teams, when both patients and their families are prone to adhere to the proposed protocol. Finally, rigorous research using outcomes important to patients, including patient-reported outcome measures, is crucial to evaluate real-life effectiveness and safety of OIT.

## Author Contributions

MG and EB conceptualized the work. FM, MG, SB, DM, BB, GL, LS, LL, US and DC drafted the manuscript. FM, MG, SB, RJ-S, DM, BB, GL, LS, LL, SR, EN, US, EB and DC were responsible for literature search, analyzed, interpreted the data and critically revised the manuscript. All authors contributed to the article and approved the submitted version.

## Funding

RJ-S acknowledges the support received by the Severo Ochoa Program (AEI/SEV-2017-0712), FSE/FEDER through the Instituto de Salud Carlos III (ISCIII; CP20/00043), The Nutricia Research Foundation (NRF-2021-13), New Frontiers in Research Fund (NFRFE-2019-00083), and SEAIC (BECA20A9). However, no significant funding source could have influenced the work in this paper.

## Conflict of Interest

The authors declare that the research was conducted in the absence of any commercial or financial relationships that could be construed as a potential conflict of interest.

## Publisher’s Note

All claims expressed in this article are solely those of the authors and do not necessarily represent those of their affiliated organizations, or those of the publisher, the editors and the reviewers. Any product that may be evaluated in this article, or claim that may be made by its manufacturer, is not guaranteed or endorsed by the publisher.

## Abbreviations

AR, adverse reaction; EAACI, European Academy of Allergy and Clinical Immunology; EoE, eosinophilic esophagitis; EPIT, epicutaneous immuntherapy; FA, food allergy; FDA, Food and Drug Administration; IL4Rα, Interleukin 4 receptor α; OIT, oral immunotherapy; QoL, quality of life; RCT, randomized controlled trials; SCIT, subcutaneous immunotherapy; sIg, allergen-specific immunoglobulin; SU, sustained unresponsiveness.
